# Uropathogenic *Escherichia coli* Metabolite-Dependent Quiescence and Persistence May Explain Antibiotic Tolerance during Urinary Tract Infection

**DOI:** 10.1128/mSphere.00055-15

**Published:** 2016-01-20

**Authors:** Mary P. Leatham-Jensen, Matthew E. Mokszycki, David C. Rowley, Robert Deering, Jodi L. Camberg, Evgeni V. Sokurenko, Veronika L. Tchesnokova, Jakob Frimodt-Møller, Karen A. Krogfelt, Karen Leth Nielsen, Niels Frimodt-Møller, Gongqin Sun, Paul S. Cohen

**Affiliations:** a Department of Cell and Molecular Biology, University of Rhode Island, Kingston, Rhode Island, USA; b Department of Biomedical and Pharmaceutical Sciences, University of Rhode Island, Kingston, Rhode Island, USA; c Department of Microbiology, University of Washington School of Medicine, Seattle, Washington, USA; d Department of Biology, Section for Functional Genomics and Center for Bacterial Stress Response (BASP), University of Copenhagen, Copenhagen, Denmark; e Department of Microbiology and Infection Control, Statens Serum Institut, Copenhagen, Denmark; f Department of Clinical Microbiology, Rigshospitalet, Copenhagen, Denmark; Swiss Federal Institute of Technology Lausanne

**Keywords:** *E. coli* quiescence, *E. coli* persistence, urinary tract infections, carbon metabolism, TCA cycle

## Abstract

In the present study, it is shown that although *Escherichia coli* CFT073, a human uropathogenic (UPEC) strain, grows in liquid glucose M9 minimal medium, it fails to grow on glucose M9 minimal medium agar plates seeded with ≤10^6^ CFU. The cells on glucose plates appear to be in a “quiescent” state that can be prevented by various combinations of lysine, methionine, and tyrosine. Moreover, the quiescent state is characteristic of ~80% of *E. coli* phylogenetic group B2 multilocus sequence type 73 strains, as well as 22.5% of randomly selected UPEC strains isolated from community-acquired urinary tract infections in Denmark. In addition, *E. coli* CFT073 quiescence is not limited to glucose but occurs on agar plates containing a number of other sugars and acetate as sole carbon sources. It is also shown that a number of *E. coli* CFT073 mini-Tn*5* metabolic mutants (*gnd*, *gdhA*, *pykF*, *sdhA*, and *zwf*) are nonquiescent on glucose M9 minimal agar plates and that quiescence requires a complete oxidative tricarboxylic acid (TCA) cycle. In addition, evidence is presented that, although *E. coli* CFT073 quiescence and persistence in the presence of ampicillin are alike in that both require a complete oxidative TCA cycle and each can be prevented by amino acids, *E. coli* CFT073 quiescence occurs in the presence or absence of a functional *rpoS* gene, whereas maximal persistence requires a nonfunctional *rpoS*. Our results suggest that interventions targeting specific central metabolic pathways may mitigate UPEC infections by interfering with quiescence and persistence.

**IMPORTANCE** Recurrent urinary tract infections (UTIs) affect 10 to 40% of women. In up to 77% of those cases, the recurrent infections are caused by the same uropathogenic *E. coli* (UPEC) strain that caused the initial infection. Upon infection of urothelial transitional cells in the bladder, UPEC appear to enter a nongrowing quiescent intracellular state that is thought to serve as a reservoir responsible for recurrent UTIs. Here, we report that many UPEC strains enter a quiescent state when ≤10^6^ CFU are seeded on glucose M9 minimal medium agar plates and show that mutations in several genes involved in central carbon metabolism prevent quiescence, as well as persistence, possibly identifying metabolic pathways involved in UPEC quiescence and persistence *in vivo*.

## Abstract (precis)

Recurrent urinary tract infections (UTIs) affect 10 to 40% of women. In up to 77% of those cases, the recurrent infections are caused by the same uropathogenic *E. coli* (UPEC) strain that caused the initial infection. Upon infection of urothelial transitional cells in the bladder, UPEC appear to enter a nongrowing quiescent intracellular state that is thought to serve as a reservoir responsible for recurrent UTIs. Here, we report that many UPEC strains enter a quiescent state when ≤10^6^ CFU are seeded on glucose M9 minimal medium agar plates and show that mutations in several genes involved in central carbon metabolism prevent quiescence, as well as persistence, possibly identifying metabolic pathways involved in UPEC quiescence and persistence *in vivo*.

## INTRODUCTION

Uncomplicated urinary tract infections (UTIs) affect about 25% of women in their lifetime, and at least 80% of those infections are caused by uropathogenic *Escherichia coli* (UPEC) (1). Recurrent UTIs affect between 10% and 40% of women (2), and in up to 77% of those cases, the recurrent infections are caused by the same UPEC strain that caused the initial infection (3, 4). UPEC infections generate annual costs in excess of two billion dollars in the United States alone, placing a significant burden on the health care system (5). Although the causes of recurrent UTI are complex (6), it appears that UPEC can bind to, enter, and replicate within superficial facet cells in the human and mouse bladder epithelium, resulting in intracellular biofilmlike communities (IBCs) (6, 7). IBCs escape from infected superficial facet cells within hours of development (6). The superficial facet cells then exfoliate, exposing underlying transitional epithelial cells, which can be infected with IBC-derived UPEC progeny (6, 8). Upon infection of urothelial transitional cells, UPEC appear to enter a nongrowing quiescent intracellular state (6, 8). These quiescent UPEC cells have been called quiescent intracellular reservoirs (QIRs) (6), and it is thought that QIRs are a major cause of recurrent UTIs (6, 8). QIRs also help to explain why antibiotics have failed to eradicate UPEC reservoirs in the bladders of mice, since quiescent UPEC may not be readily affected by antibiotics (6, 8).

The quiescence of QIRs and their insensitivity to antibiotics is reminiscent of the persister state (8–11). Persister cells are dormant cells formed in normal microbial populations as small subpopulations that are highly tolerant to antibiotics but upon regrowth in the absence of antibiotics regain full sensitivity (11). Persisters appear to play a major role in the ability of chronic infections to withstand antibiotic treatment (11). In the present study, we report that when inocula of ≤10^6^ CFU of *E. coli* CFT073, the prototypic UPEC strain, as well as ~80% of phylogenetic group B2 multilocus sequence type 73 (ST73) strains, of which *E. coli* CFT073 is a member, are plated on M9 minimal agar plates containing glucose as the sole carbon and energy source, they appear to enter a “quiescent” state and that mutations in specific metabolic genes appear to prevent that state. In addition, we show that *E. coli* CFT073 quiescence also occurs in the presence of a number of other sugars and acetate as sole carbon sources and that a complete tricarboxylic acid (TCA) cycle is required both for the generation of *E. coli* CFT073 quiescent cells on glucose plates and for the formation of persister cells generated in liquid glucose minimal medium in the presence of ampicillin.

## RESULTS

### *E. coli* CFT073, a UPEC strain, and *E. coli* Nissle 1917, a closely related probiotic strain, grow in liquid glucose M9 minimal medium but fail to grow on glucose M9 minimal medium agar plates.

We were attempting to determine which colicins and microcins are active against the sequenced *E. coli* CFT073, a phylogenetic group B2 multilocus sequence type 73 (ST73) UPEC strain (20, 21), and the closely related ST73 probiotic strain, *E. coli* Nissle 1917 (22). As expected, we found that after overnight incubation at 37°C, both strains formed lawns of growth on 0.2% glucose M9 minimal medium agar plates (hereinafter called glucose plates) when either 10^7^ or 10^8^ CFU was plated from an overnight 0.4% glucose M9 minimal medium liquid culture. Unexpectedly, however, when 10^6^ CFU or fewer were plated on glucose plates, they failed to form lawns or colonies after 24 h and 48 h of incubation at 37°C. This result was unusual in that a number of human commensal *E. coli* strains, including MG1655, HS, F-18, EFC1, and EFC2 (Table 1), and the O157:H7 strain *E. coli* EDL933 (Table 1) tested at 10^5^ CFU grew as lawns on the glucose plates after 24 h at 37°C, and viable counts could be determined for each strain on glucose plates (not shown). That all the *E. coli* strains tested for growth on glucose plates are spontaneous streptomycin-resistant mutants (Table 1) but only *E. coli* CFT073 and *E. coli* Nissle 1917 failed to form lawns on glucose plates seeded with ≤10^6^ CFU makes it highly unlikely that point mutations in ribosomal proteins play a role in the observed lack of growth.

**TABLE 1:** Bacterial strains

*E. coli* strain	Genotype/phenotype	Designation in text	Source or reference
CFT073 Str^r^	Spontaneous streptomycin-resistant mutant of CFT073, has 5-bp duplication in *rpoS*	CFT073	53
CFT073 Str^r^ mini-Tn*5* Km*::gdhA*	Mini-Tn*5* Km glutamate dehydrogenase mutant of CFT073 Str^r^	CFT073 *gdhA*	This study
CFT073 Str^r^ mini-Tn*5* Km*::gnd*	Mini-Tn*5* Km 6-phosphogluconate dehydrogenase mutant of CFT073 Str^r^	CFT073 *gnd*	This study
CFT073 Str^r^ mini-Tn*5* Km*::pykF*	Mini-Tn*5* Km pyruvate kinase mutant of CFT073 Str^r^	CFT073 *pykF*	This study
CFT073 Str^r^ mini-Tn*5* Km*::sdhA*	Mini-Tn*5*::Km flavoprotein subunit of succinate dehydrogenase mutant of CFT073 Str^r^	CFT073 *sdhA*	This study
CFT073 Str^r^ mini-Tn*5* Km*::zwf*	Mini-Tn*5*::Km glucose-6-phosphate dehydrogenase mutant of CFT073 Str^r^	CFT073 *zwf*	This study
Wild-type CFT073	Original clinical isolate	CFT073 original clinical isolate	34
Nissle 1917 Str^r^	Spontaneous streptomycin-resistant mutant of Nissle 1917	Nissle 1917	57
MG1655 Str^r^	Spontaneous streptomycin-resistant mutant of MG1655	MG1655	54
HS Str^r^	Spontaneous streptomycin-resistant mutant of HS	HS	55
EFC1 Str^r^	Spontaneous streptomycin-resistant mutant of EFC1	EFC1	55
EFC2 Str^r^	Spontaneous streptomycin-resistant mutant of EFC2	EFC2	55
F-18 Str^r^ Nal^r^	Spontaneous streptomycin- and nalidixic acid-resistant mutant of F-18	F-18	56
EDL933 Str^r^	Spontaneous streptomycin-resistant mutant of EDL933	EDL933	54
ATM161	Host for pUT, which contains the mini-Tn*5* Km transposon (kanamycin resistance)	ATM161	17

**FIG 1:**
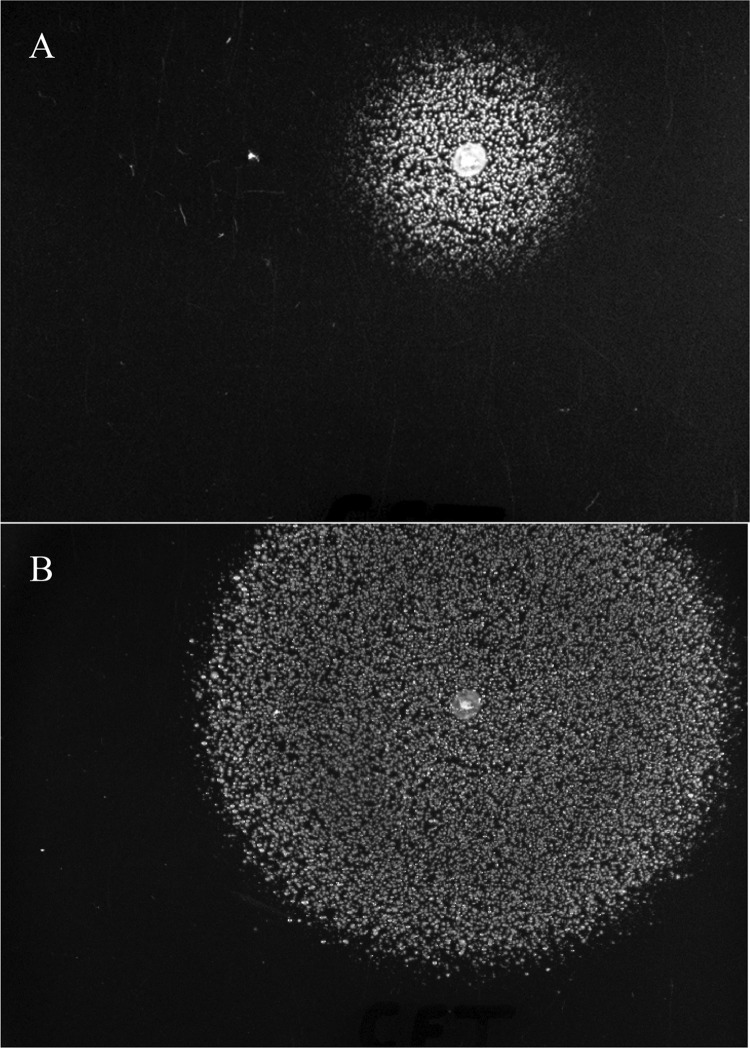
*E. coli* CFT073 quiescence on glucose plates. A 0.2% glucose plate was seeded with 10^5^ CFU of *E. coli* CFT073 (see Materials and Methods). (A) Sixty minutes after seeding the plate, a colony of *E. coli* MG1655, grown on a glucose plate, was transferred to the plate seeded with *E. coli* CFT073, using a toothpick; the plate was then incubated at 37°C for 24 h. (B) The same plate, incubated for 48 h. Note that *E. coli* CFT073 only grows around the picked *E. coli* MG1655. Although not shown, *E. coli* Nissle 1917 undergoes quiescence on glucose plates identically.

Picking colonies of *E. coli* MG1655 grown on glucose plates onto glucose plates seeded with 10^5^ or 10^6^ CFU of *E. coli* CFT073 or *E. coli* Nissle 1917, using toothpicks, resulted in *E. coli* CFT073 and *E. coli* Nissle 1917 growth around the picked colonies but not anywhere else on the plates after incubation for 24 h at 37°C (Fig. 1A) (using toothpicks to transfer colonies in this manner is a normal procedure used in colicin and microcin testing). No growth was observed around picked *E. coli* MG1655 colonies grown on glucose plates that had not been seeded with *E. coli* CFT073 or *E. coli* Nissle 1917 (not shown). These results suggested that picked *E. coli* MG1655 was secreting a molecule(s) (hereinafter called the MG1655 stimulus) as it grew on glucose plates that was either preventing cell death of *E. coli* CFT073 and *E. coli* Nissle 1917 or preventing them from entering a quiescent state. Incubating the plates that had been seeded with 10^5^ CFU of *E. coli* CFT073 or *E. coli* Nissle 1917 for 48 h at 37°C resulted in much larger regions of growth around the picked *E. coli* MG1655 colonies than at 24 h (Fig. 1B), suggesting that cells could be stimulated to grow after 24 h of incubation and thereby favoring the quiescence hypothesis and suggesting either that the MG1655 stimulus was diffusible and active at a very low concentration or that growing *E. coli* CFT073 and *E. coli* Nissle 1917 also secreted the stimulus. Also, when glucose plates seeded with 10^5^ or 10^6^ CFU of *E. coli* CFT073 or *E. coli* Nissle 1917 were incubated for 24 h at 37°C prior to picking *E. coli* MG1655 to those plates, growth of *E. coli* CFT073 and *E. coli* Nissle 1917 was observed surrounding the picked *E. coli* MG1655 colonies after an additional 24-h incubation at 37°C (not shown). Therefore, many of the *E. coli* CFT073 and *E. coli* Nissle 1917 cells were alive but quiescent on glucose plates for at least 24 h. Importantly, quiescence is not observed when Difco Bacto agar is used in glucose plates instead of Difco noble agar, suggesting that impurities in the former allow growth. It should be noted that at this time, we do not know whether it is live or dead *E. coli* MG1655 cells that are the source of the stimulus, as the picked cells grow on the glucose plates. Also, it should be mentioned that *E. coli* CFT073 and *E. coli* Nissle 1917 quiescence is not dependent on using 0.2% glucose M9 minimal medium agar plates, since identical results were obtained using 0.4% glucose M9 minimal medium agar plates (not shown).

### Testing additional *E. coli* strains for quiescence on glucose plates.

*E. coli* strains can be separated into four major phylogenetic groups (A, B1, B2, and D) and two additional phylogenetic groups that have recently been defined, phylogenetic group AxB1, containing strains that derive most of their ancestry from A and B1, and phylogenetic group ABD, containing a heterogeneous set of strains with multiple sources of ancestry (23). Thirty *E. coli* strains representing various multilocus sequence types (ST) of the 6 phylogenetic groups were grown in liquid glucose M9 minimal medium, and the 30 strains were tested for the ability to grow on glucose plates seeded with inocula of 10^5^ CFU and to respond to the MG1655 stimulus. The strains used included two ST10 and two ST453 strains from phylogenetic group A; two ST58, two ST410, and two ST101 strains from phylogenetic group B1; two ST73, two ST95, and two ST131 strains from phylogenetic group B2; two ST69, two ST354, and two ST648 strains from phylogenetic group D; two ST90 and two ST642 strains from phylogenetic group AxB1; and two ST62 and two ST117 strains from phylogenetic group ABD. Of the 30 strains, 2 failed to grow on glucose plates and those strains responded to the MG1655 stimulus. The 2 strains that failed to grow on glucose plates were ST73 strains. ST73 is a very common UPEC lineage, accounting for 11% and 16.6% of UPEC isolated from patients in 2 recent studies (24, 25). Importantly, *E. coli* CFT073 and *E. coli* Nissle 1917 are also ST73 strains.

### Testing additional ST73 strains for quiescence on glucose plates.

Since it appeared that quiescence on glucose agar plates might be characteristic of the ST73 lineage, 40 additional ST73 strains were tested for the ability to grow on glucose plates seeded with inocula of 10^5^ CFU and to respond to the MG1655 stimulus. Two of the strains failed to grow overnight in liquid glucose M9 minimal medium, but of the 38 strains that grew, 30 (78.9%) failed to grow on glucose plates but responded to the MG1655 stimulus. Therefore, the vast majority of ST73 strains, a major UPEC lineage (24, 25), are quiescent on glucose plates.

### Testing 40 UPEC strains isolated from community-acquired UTIs in Denmark for quiescence on glucose plates.

Forty randomly selected UPEC strains isolated from community-acquired UTIs in Denmark were tested for the ability to grow on glucose plates seeded with inocula of 10^5^ CFU and to respond to the MG1655 stimulus. Of the 40 UPEC strains tested, all grew overnight in liquid glucose M9 minimal medium, but 9 failed to grow on glucose plates (22.5%) unless stimulated to do so by the MG1655 stimulus. Three of the 9 strains that failed to grow on glucose plates were ST73 strains (5 of the 40 UPEC strains tested [12.5%] were ST73), and 3 were ST141 strains (3 of the 40 UPEC strains tested were ST141 strains [7.5%], a group not represented in the original 30 strains tested). The 3 remaining strains that failed to grow on glucose plates (ST104, ST394, and ST998) were not represented in the original 30 strains tested, and each was represented only once among the 40 UPEC strains tested (2.5% each). It therefore appears that the inability to grow on glucose plates and yet respond to the MG1655 stimulus is not limited to the ST73 group.

### The inability of *E. coli* CFT073 to grow on minimal agar plates is not limited to glucose as sole carbon source.

**FIG 2:**
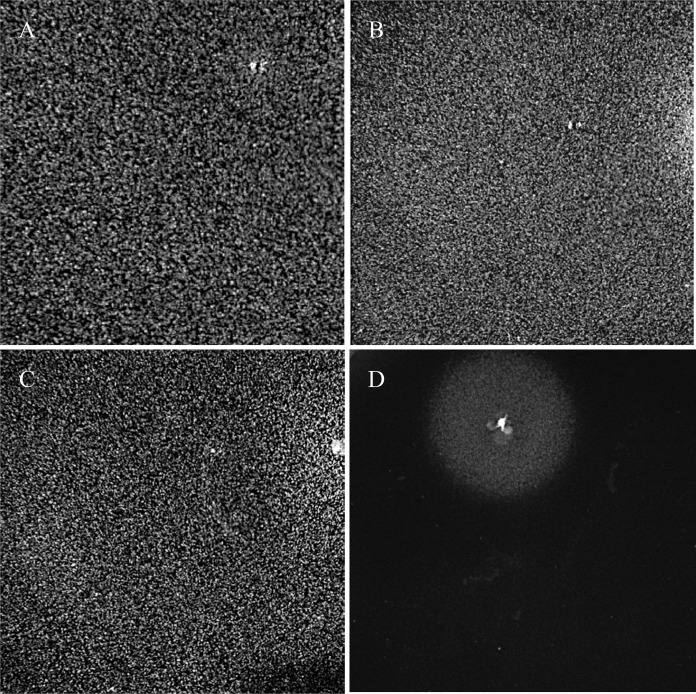
*E. coli* CFT073 nonquiescence on glycerol, ribose, and xylose plates. Glucose, glycerol, ribose, and xylose plates (0.2% each) were seeded with 10^5^ CFU of *E. coli* CFT073 grown overnight in liquid M9 minimal medium containing their respective sugars (0.4%). Sixty minutes after seeding the plates, a colony of *E. coli* MG1655, grown on a glucose plate, was transferred to a glucose plate, using a toothpick. Plates were incubated at 37°C for 24 h. (A) Glycerol; (B) ribose; (C) xylose; (D) glucose.

*E. coli* CFT073 was tested for the ability of 10^5^ CFU to grow on M9 minimal medium agar plates containing 0.2% acetate, arabinose, fructose, fucose, galactose, gluconate, glycerol, *N*-acetylglucosamine, maltose, mannose, ribose, and xylose as sole carbon sources. *E. coli* CFT073 grew overnight in liquid M9 minimal medium containing each carbon source, but inocula of 10^5^ CFU only grew as lawns on agar plates containing glycerol, ribose, and xylose as sole carbon sources (Fig. 2). On those plates where *E. coli* CFT073 failed to grow, it responded to the MG1655 stimulus.

### Human urine, a cocktail mimicking the amino acid composition of human urine, and a cocktail mimicking amino acids present in a concentrated *E. coli* MG1655 culture supernatant prevent *E. coli* CFT073 quiescence on glucose plates.

**FIG 3:**
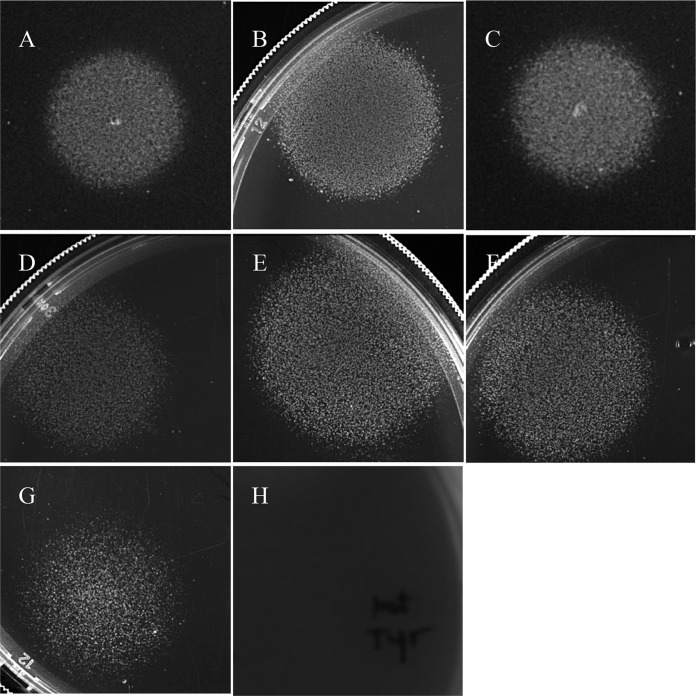
Prevention of quiescence by human urine and amino acids. Glucose (0.2%) plates were seeded with 10^5^ CFU of *E. coli* CFT073, and 5-µl amounts of the following mixtures were spotted onto the plates: (A) 50-fold-concentrated *E. coli* MG1655 culture supernatant; (B) amino acid cocktail mimicking the amino acid concentrations in the 50-fold-concentrated *E. coli* MG1655 culture supernatant; (C) human urine; (D) amino acid cocktail mimicking the amino acid concentrations in human urine (Table 3); (E) lysine, methionine, and tyrosine (1.0 mM each); (F) lysine and methionine (1.0 mM each); (G) lysine and tyrosine (1.0 mM each); and (H) methionine and tyrosine (1.0 mM each). Plates were incubated at 37°C for 24 h. Although not shown, the results for *E. coli* Nissle 1917 were essentially identical.

We normally pick a colony of *E. coli* MG1655 to a glucose plate as a source of the MG1655 stimulus, suggesting that the stimulus is secreted on the plate as *E. coli* MG1655 grows. However, no stimulus activity was found when 5 µl or 20 µl of a cell-free supernatant derived from an overnight liquid glucose M9 minimal medium culture of *E. coli* MG1655 was placed on a glucose agar plate seeded with 10^5^ CFU of *E. coli* CFT073 or *E. coli* Nissle 1917. Stimulus activity was observed when 5 µl of a cell-free supernatant derived from a 50-fold-concentrated *E. coli* MG1655 culture that had been incubated overnight at 37°C (see Materials and Methods) was placed on a glucose agar plate seeded with 10^5^ CFU of *E. coli* CFT073 (Fig. 3A). Analysis of one such *E. coli* MG1655 cell-free supernatant revealed the presence of a number of unknown small molecules and 14 amino acids (Table 2). Importantly, 5 µl of an amino acid cocktail identical in composition to the amino acids in the 50-fold-concentrated *E. coli* MG1655 supernatant displayed stimulus activity similar to that of 5 µl of the 50-fold-concentrated supernatant (Fig. 3B). A cell-free supernatant derived from a 50-fold-concentrated *E. coli* CFT073 culture was nearly identical to the *E. coli* MG1655 supernatant in amino acid composition but additionally contained aspartic acid (Table 2). As expected, 5 µl of the *E. coli* CFT073 cell-free supernatant also displayed stimulus activity on glucose plates seeded with 10^5^ CFU of *E. coli* CFT073. Perhaps even more importantly, 5 µl of sterile filtered human urine collected from one of us and 5 µl of a cocktail mimicking the amino acid composition of human urine (Table 2) (26) both displayed stimulus activity on glucose plates seeded with 10^5^ CFU of *E. coli* CFT073 (Fig. 3C and D).

**TABLE 2:** Free-amino-acid composition of 50-fold-concentrated *E. coli* MG1655 and *E. coli* CFT073 supernatants and human urine

Amino acid	Amt (µM) of amino acid in^a^:		
	*E. coli* MG1655 supernatant^b^	*E. coli* CFT073 supernatant^b^	Human urine^c^
Alanine	353	383	3,350
Arginine	—	—	205
Aspartic acid	—	22	—
Cysteine	—	—	1,110
Glutamic acid	360	848	—
Glycine	11	86	21,200
Histidine	—	—	9,470
Isoleucine	90	170	478
Leucine	56	149	382
Lysine	7,472	4,059	4,480
Methionine	59	37	171
Phenylalanine	99	187	626
Proline	144	116	—
Serine	64	70	4,000
Threonine	201	461	2,430
Tryptophan	146	312	—
Tyrosine	13	52	1,060
Valine	229	748	349

^a^ —, amino acid was not present in the preparation.

^b^ See Materials and Methods for details.

^c^ Average values of samples from 39 women (26).

### Lysine, methionine, and tyrosine are involved in preventing quiescence.

One millimolar solutions of each of the 20 standard l-amino acids were prepared, and 5 µl of each was tested on glucose plates seeded with 10^5^ CFU of *E. coli* CFT073. None displayed stimulus activity. However, when single amino acids were omitted from the cocktails, testing 5 µl of the cocktails mimicking the concentrations of amino acids in urine (Table 2) and in the 50-fold-concentrated *E. coli* MG1655 supernatant (Table 2) revealed that cocktails missing lysine, methionine, and tyrosine failed to stimulate. Furthermore, although 5 µl of 1.0 mM lysine alone, 1.0 mM methionine alone, and 1.0 mM tyrosine alone failed to stimulate (not shown), 5 µl of mixtures of 1.0 mM each of lysine, methionine, and tyrosine (Fig. 3E) were about as stimulatory for growth of *E. coli* CFT073 on glucose plates as either the *E. coli* MG1655 amino acid cocktail (Fig. 3B) or the amino acid cocktail mimicking human urine (Fig. 3D). Five microliters of mixtures of 1.0 mM each of d-lysine, d-methionine, and d-tyrosine failed to stimulate (not shown), demonstrating the importance of the l- forms of the 3 amino acids in preventing quiescence. Mixtures of 1.0 mM each of lysine and methionine (Fig. 3F) and 1.0 mM each of lysine and tyrosine (Fig. 3G) also stimulated *E. coli* CFT073 growth on glucose plates, although the stimulation of *E. coli* CFT073 growth by the mixture of lysine and tyrosine was minimal (Fig. 3G). A mixture of 1.0 mM each of methionine and tyrosine failed to stimulate *E. coli* CFT073 growth (Fig. 3H). In summary, *E. coli* MG1655 is not required to provide the stimulus that prevents *E. coli* CFT073 quiescence; a mixture of lysine, methionine, and tyrosine, found in 50-fold-concentrated *E. coli* MG1655 supernatants, is just as effective.

### *E. coli* CFT073 and *E. coli* Nissle 1917 but not *E. coli* MG1655 generate high levels of persister cells in liquid glucose M9 minimal medium.

**FIG 4:**
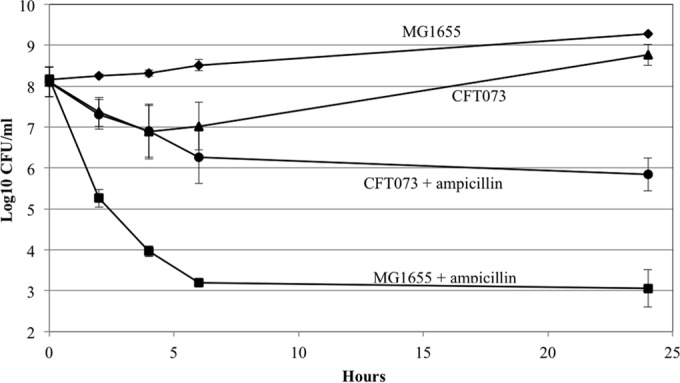
*E. coli* CFT073 and *E. coli* MG1655 persistence. Cultures were grown overnight in 0.4% glucose M9 minimal medium as described in Materials and Methods. Persister cell assays were performed as described in Materials and Methods. ▲, *E. coli* CFT073; ●, *E. coli* CFT073 plus ampicillin; ♦, *E. coli* MG1655; ■, *E. coli* MG1655 plus ampicillin. Bars representing standard errors of the means of counts from 2 independent experiments are presented for each time point. At 24 h, the approximately 1,000-fold difference between the counts of *E. coli* CFT073 persisters and *E. coli* MG1655 persisters in the presence of ampicillin is statistically significant (*P* = 0.0052).

Persister cells are dormant cells formed in normal microbial populations as small subpopulations (10^−3^ to 10^−4^%) that are highly tolerant to antibiotics but, upon regrowth in the absence of antibiotics, regain full sensitivity (11). Persister cells appear to play a role in the ability of bacteria causing chronic infections to withstand antibiotic treatment (11). Because *E. coli* CFT073 becomes quiescent on glucose plates and quiescence is reminiscent of persistence, we wondered whether *E. coli* CFT073 would generate a high level of persister cells in liquid glucose M9 minimal medium. Overnight cultures of *E. coli* CFT073 grown on 0.4% glucose M9 minimal medium were diluted 20-fold into fresh 0.2% glucose M9 minimal medium (*A*_600_ of 0.1, ~10^8^ CFU/ml) containing or lacking ampicillin (100 µg/ml), and viable counts were followed for 24 h at 37°C (Fig. 4). During the first 4 h of incubation, the viable counts in the *E. coli* CFT073 cultures decreased 10-fold in both the presence and absence of ampicillin, i.e., from 10^8^ CFU/ml to 10^7^ CFU/ml. No further cell death occurred between 4 h and 6 h in the absence of ampicillin, and by 24 h, *E. coli* CFT073 had grown to almost 10^9^ CFU/ml (Fig. 4). In contrast, between 4 h and 6 h in the presence of ampicillin, the viable counts decreased almost an additional 10-fold, to about 10^6^ CFU/ml, in the *E. coli* CFT073 cultures (Fig. 4). However, there was little further *E. coli* CFT073 death in the presence of ampicillin between 6 h and 24 h (Fig. 4), suggesting the possibility that the survivors (~0.7%) at 24 h might be persisters. Therefore, at 24 h, *E. coli* CFT073 cultures containing ampicillin were centrifuged, washed free of the antibiotic, resuspended in LB broth, and grown at 37°C for 2.25 h to 10^8^ CFU/ml, at which time ampicillin was added (100 µg/ml). Four hours later, viable counts in the *E. coli* CFT073 LB broth cultures had dropped to 10^4^ CFU/ml, suggesting that the vast majority of cells that survived ampicillin treatment in liquid glucose M9 minimal medium were indeed persister cells, still fully sensitive to ampicillin. Much like *E. coli* CFT073, *E. coli* Nissle 1917 generated a high level of persister cells in liquid glucose minimal medium, i.e., about 2.6% at 24 h (Table 3), and the cells were still sensitive to ampicillin, as described above.

**TABLE 3:** *E. coli* CFT073 persister cells relative to persister cells for other *E. coli* strains

*E. coli* strain	No. of experiments	% of persister cells ± SEM^a^	Persister cell ratio of CFT073 and indicated strain^a^	*P* value^b^
CFT073	7	0.71 ± 0.19	−	
CFT073 *gnd*	2	0.34 ± 0.18	1.65	0.44
CFT073 *pykF*	2	2.37 ± 0.18	0.23	0.075
CFT073 *zwf*	2	0.29 ± 0.29	1.45	0.43
Nissle 1917	2	2.64 ± 1.22	0.21	0.16
True wild-type CFT073	2	(2.80 ± 1.1) × 10^−4^	1,160	0.025

^a^ The percentage of persister cells was calculated by dividing the viable count at 24 h by the viable count at time zero times 100. The value for *E. coli* CFT073 persister cells relative to a specific *E. coli* strain was calculated by dividing the percentage of persister cells generated by *E. coli* CFT073 at 24 h in the experiments for each specific strain by the percentage of persister cells generated by that specific strain at 24 h in those experiments.

^b^ A *P* value of <0.05 using the two-tailed Student’s *t* test is considered to be statistically significant.

Unlike *E. coli* CFT073 and *E. coli* Nissle 1917, when *E. coli* MG1655 overnight cultures were diluted 20-fold into fresh 0.2% glucose M9 minimal medium, viable counts increased immediately in the absence of ampicillin, and in the presence of ampicillin, decreased continuously for 6 h to a level of about 10^3^ CFU/ml (10^−3^%) and remained at that level at 24 h (Fig. 4). The *E. coli* MG1655 survivors in cultures containing ampicillin were also persister cells, i.e., when regrown in LB broth without ampicillin, they regained sensitivity. Therefore, when grown in liquid glucose M9 minimal medium, *E. coli* CFT073 and *E. coli* Nissle 1917 cultures generated about 1,000-fold more persister cells than *E. coli* MG1655 cultures.

### *E. coli* CFT073 generates a low level of persister cells in liquid glucose M9 minimal medium containing amino acids.

**FIG 5:**
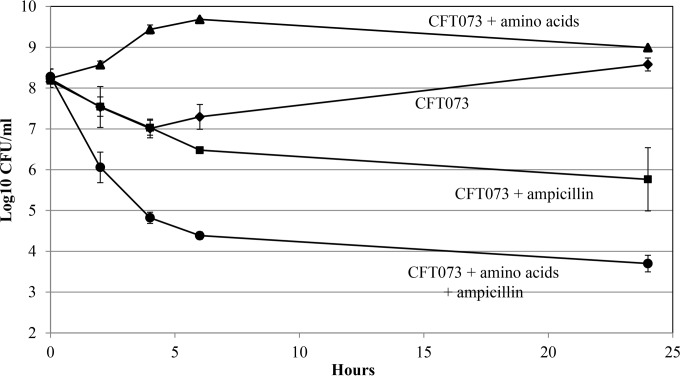
*E. coli* CFT073 persistence in the presence of amino acids. Cultures were grown in 0.4% glucose M9 minimal medium as described in Materials and Methods and diluted 20-fold into 0.2% glucose M9 minimal medium either containing or lacking a mixture of the 20 standard l-amino acids, each at 100 µg/ml, and containing or lacking ampicillin (100 µg/ml). ♦, *E. coli* CFT073; ■, *E. coli* CFT073 plus ampicillin; ▲, *E. coli* CFT073 plus amino acids; ●, *E. coli* CFT073 plus amino acids plus ampicillin. Bars representing standard errors of the means of counts from 2 independent experiments are presented for each time point. At 6 h and 24 h, the approximately 100-fold differences between *E. coli* CFT073 persisters and *E. coli* MG1655 persisters in the presence of ampicillin are statistically significant (*P* = 0.002 and *P* = 0.05, respectively).

Since amino acids reversed *E. coli* CFT073 quiescence on glucose plates, we were interested in determining whether the addition of a mixture of the 20 standard l-amino acids (100 µg/ml each) to cultures of *E. coli* CFT073 grown in glucose M9 minimal medium in the absence of amino acids would generate fewer persister cells. As shown by the results in Fig. 5, in the presence of the amino acid mixture and absence of ampicillin, *E. coli* CFT073 viable counts increased immediately, reaching stationary phase within 4 h. Importantly, in the presence of both the amino acid mixture and ampicillin, *E. coli* CFT073 viable counts decreased continuously for 24 h, to a level of about 5 × 10^3^ CFU/ml (Fig. 5). That the survivors at 24 h were persister cells was shown by the fact that when regrown in LB broth without ampicillin, they regained sensitivity, as described above. In the absence of amino acids and presence of ampicillin, *E. coli* CFT073 persister cells were again generated, at a level of about 10^6^ CFU/ml (Fig. 5). Therefore, in the presence of amino acids, about 100-fold fewer *E. coli* CFT073 persister cells were generated than in their absence (Fig. 5).

### Isolation and characterization of *E. coli* CFT073 mini-Tn*5* mutants that grow on glucose plates.

Since *E. coli* CFT073 grows overnight in liquid glucose M9 minimal medium but not on glucose plates, we thought it possible that the expression of one or more genes on glucose plates but not in liquid glucose cultures might be responsible. If so, knockout of the responsible gene(s) would result in growth on glucose plates. Therefore, *E. coli* CFT073 mini-Tn*5* Km (kanamycin) mutants were generated by random insertional mutagenesis (see Materials and Methods), and any mutant that grew as lawns on glucose plates was confirmed by transferring the insertion into a fresh *E. coli* CFT073 background and retesting it for growth on glucose plates (see Materials and Methods).

Five confirmed nonquiescent mini-Tn*5* Km mutants were isolated from approximately 2,000 mutants tested. *E. coli* CFT073 and the mini-Tn*5* Km mutants were grown overnight in liquid glucose M9 minimal medium, and viable counts were made on both glucose plates and LB agar plates. As expected, *E. coli* CFT073 assayed from the overnight cultures failed to grow on the glucose plates when inocula of ≤10^6^ CFU were plated, but when assayed on LB agar, viable counts showed that the cultures contained ~10^9^ CFU/ml. In contrast, when assayed on either glucose plates or LB agar plates, the 5 mini-Tn*5* Km mutant cultures each contained about ~10^9^ CFU/ml. It therefore appears that the mini-Tn*5* Km insertion in each of the 5 genes completely prevented quiescence on glucose plates.

**FIG 6:**
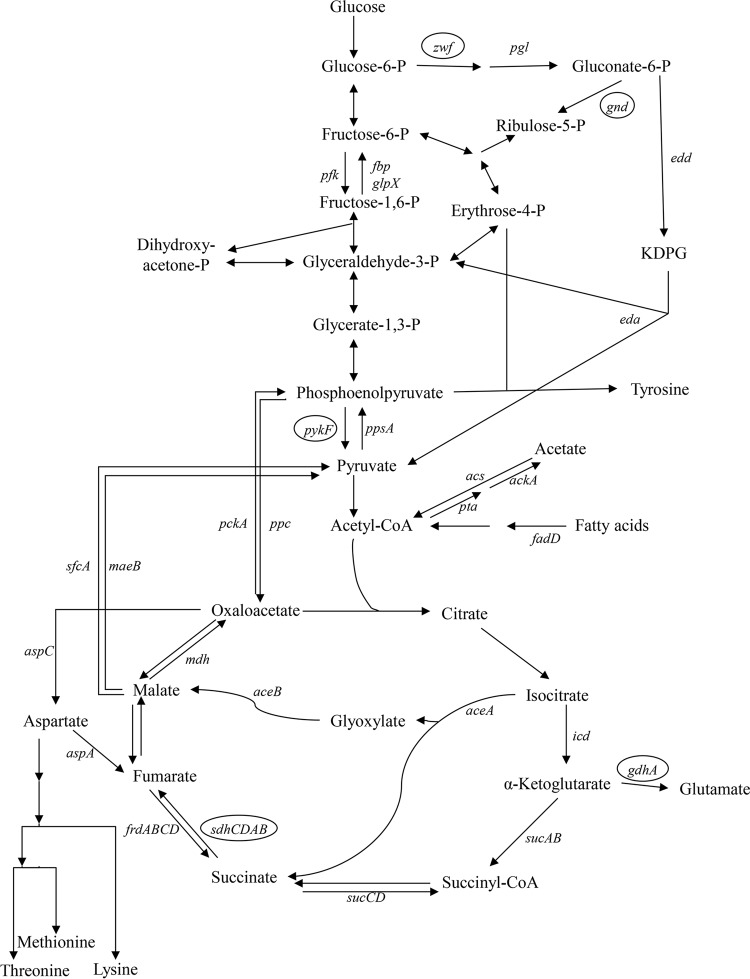
Diagram of *E. coli* central carbon metabolism. Arrows indicate the physiological directions of the reactions. Genes encoding the enzymes for each reaction are listed beside each reaction. Mini-Tn*5* Km insertions in *E. coli* CFT073 genes that result in nonquiescence on glucose plates are circled. P, phosphate; KDPG, 2-keto-3-deoxy-6-phosphogluconate.

The mini-Tn*5* Km insertions that resulted in mutants able to grow on glucose plates after transfer into a fresh *E. coli* CFT073 background were in the *sdhA*, *gnd*, *zwf*, *pykF*, and *gdhA* genes (Fig. 6). (i) *sdhA* encodes the succinate-binding flavoprotein subunit of succinate dehydrogenase (27). As a consequence of the mutation, the *E. coli* CFT073 *sdhA* mutant fails to grow on succinate as a sole carbon source, but it grows normally on glucose. (ii) *gnd* encodes 6-phosphogluconate dehydrogenase, which functions in the oxidative branch of the pentose phosphate pathway to synthesize ribulose-5-phosphate from 6-phosphogluconate (28, 29). Ribulose-5-phosphate is an essential precursor in the synthesis of FAD, nucleotides, and lipopolysaccharides (LPS). (iii) *zwf* encodes glucose-6-phosphate dehydrogenase, which functions in the oxidative branch of the pentose phosphate pathway and the Entner-Doudoroff pathway when *E. coli* is grown on glucose (28, 29). (iv) *pykF* encodes pyruvate kinase, which converts phosphoenolpyruvate to pyruvate in the Embden-Meyerhof-Parnas pathway (30). (v) *gdhA* encodes glutamate dehydrogenase, which catalyzes the amination of α-ketoglutarate to glutamate (31).

It might be argued that the mini-Tn*5* Km insertions in the identified genes are not the cause of nonquiescence but, rather, that nonquiescence is caused by downstream polarity effects. Indeed, the mini-Tn*5* Km transposon used in the present study has strong transcription termination sequences flanking both ends of the kanamycin resistance gene (17). However, the intergenic number of nucleotides and nucleotide sequences between *gnd* and the immediately downstream gene *ugd*, between *pykF* and the immediately downstream gene *lpp*, and between *zwf* and the immediately downstream gene *edd* are identical in *E. coli* MG1655 and *E. coli* CFT073 (GenBank accession numbers U00096.3 and AE014075.1) (21, 32). Moreover, in *E. coli* MG1655 and, therefore, in *E. coli* CFT073, there is a strong presumptive promoter between *gnd* and *ugd* (33), and in *E. coli* MG1655 and, therefore, in *E. coli* CFT073, both *lpp* and *edd* have their own experimentally identified promoters (33). It is therefore highly likely that nonquiescence is caused by interrupting *gnd*, *pykF*, and *zwf* and not by downstream polarity effects. Also, in *E. coli* CFT073, *gdhA* is immediately upstream from c2163, which is transcribed in the opposite direction to *gdhA* (21), making it highly unlikely that nonquiescence caused by the insertion in *gdhA* is due to downstream polarity. Finally, although in *E. coli* MG1655, the number of nucleotides between the end of the *sdhCDAB* operon and the beginning of the immediately downstream *sucABCD* operon is 241 nucleotides less than that in *E. coli* CFT073 (GenBank accession numbers U00096.3 and AE014075.1) (31, 32), the experimentally identified *E. coli* MG1655 *sucABCD* promoter is identical in sequence to the presumptive *E. coli* CFT073 *sucABCD* promoter, and the nucleotide sequence between the 3′ end of the promoter and the start of *sucA* transcription is identical in both strains (21, 32, 33). It is therefore highly unlikely that nonquiescence caused by the insertion in *sdhA* is due to a downstream polarity effect on the *sucABCD* operon in *E. coli* CFT073.

### Of the 5 mini-Tn*5* Km mutants, only *E. coli* CFT073 *sdhA* and *gdhA* mutants generate low levels of persister cells.

**FIG 7:**
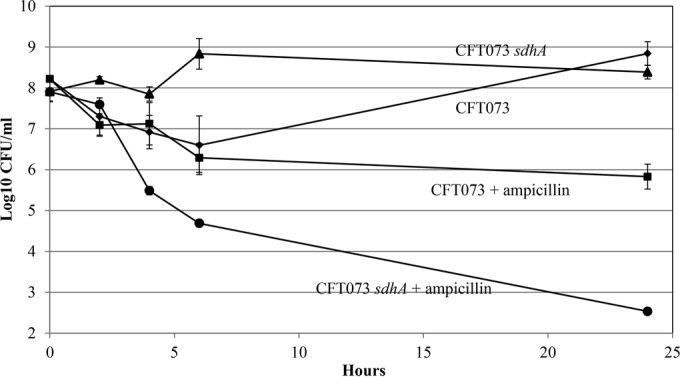
*E. coli* CFT073 *sdhA* persistence. Cultures were grown overnight in 0.4% glucose M9 minimal medium, and persister cell assays were performed as described in Materials and Methods. ♦, *E. coli* CFT073; ■, *E. coli* CFT073 plus ampicillin; ▲, *E. coli* CFT073 *sdhA*; ●, *E. coli* CFT073 *sdhA* plus ampicillin. Bars representing standard errors of the means of counts from 4 independent experiments are presented for each time point. At 24 h, the approximately 2,000-fold difference between *E. coli* CFT073 persisters and *E. coli* CFT073 *sdhA* persisters in the presence of ampicillin is statistically significant (*P* < 0.001).

**FIG 8:**
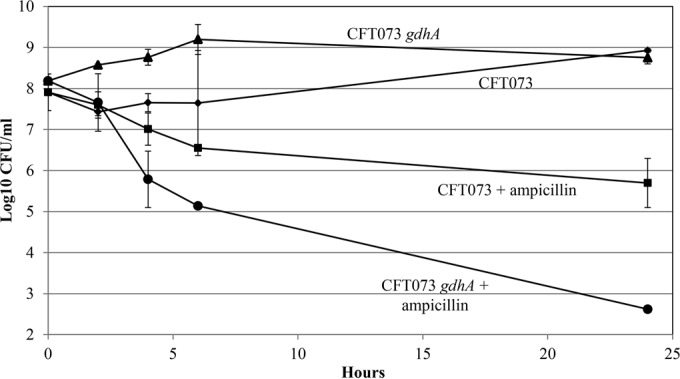
*E. coli* CFT073 *gdhA* persistence. Cultures were grown overnight in 0.4% glucose M9 minimal medium, and persister cell assays were performed as described in Materials and Methods. ♦, *E. coli* CFT073; ■, *E. coli* CFT073 plus ampicillin; ▲, *E. coli* CFT073 *gdhA*; ●, *E. coli* CFT073 *gdhA* plus ampicillin. Bars representing standard errors of the means of counts from 3 independent experiments are presented for each time point. At 24 h, the approximately 2,000-fold difference between *E. coli* CFT073 persisters and *E. coli* CFT073 *gdhA* persisters in the presence of ampicillin is statistically significant (*P* < 0.001).

Since the 5 *E. coli* CFT073 mini-Tn*5* Km mutants were nonquiescent on glucose plates, we wondered whether they would generate fewer persister cells than *E. coli* CFT073 in glucose M9 minimal medium. Indeed, the *E. coli* CFT073 *sdhA* and *gdhA* mutants, which began growth shortly after dilution of overnight cultures into fresh glucose M9 minimal medium, generated only about 5 × 10^2^ persister cells/ml, i.e., about 2,000-fold fewer persister cells in the presence of ampicillin than wild-type *E. coli* CFT073 (Fig. 7 and Fig. 8). In contrast, the *E. coli* CFT073 *gnd*, *pykF*, and *zwf* mutants generated about the same levels of persister cells as *E. coli* CFT073 (Table 3), suggesting that quiescence and persistence are not identical phenomena.

### *E. coli* CFT073 persistence and quiescence require a complete TCA cycle.

**FIG 9:**
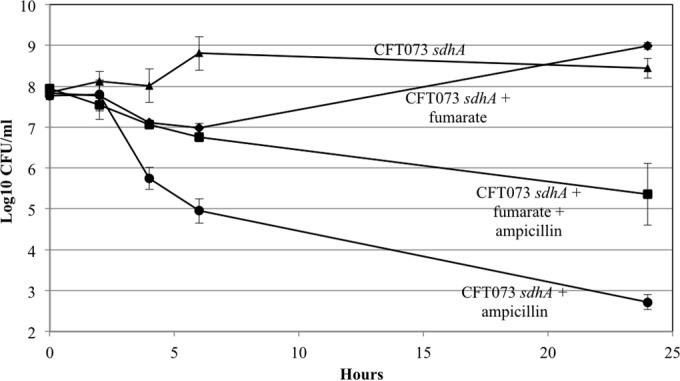
*E. coli* CFT073 *sdhA* persistence in the presence of fumarate. Cultures were grown overnight in 0.4% glucose M9 minimal medium containing or lacking disodium fumarate (200 µg/ml) and then diluted 20-fold into 0.2% glucose M9 minimal medium containing or lacking fumarate, and persister cell assays were performed as described in Materials and Methods. ▲, *E. coli* CFT073 *sdhA*; ●, *E. coli* CFT073 *sdhA* plus ampicillin; ♦, *E. coli* CFT073 *sdhA* plus fumarate; ■, *E. coli* CFT073 *sdhA* plus fumarate plus ampicillin. Bars representing standard errors of the means of counts from 2 independent experiments are presented for each time point. At 6 h and 24 h, the differences between *E. coli* CFT073 *sdhA* persisters in the presence and absence of fumarate are statistically significant (*P* = 0.013 and *P* = 0.046, respectively).

Succinate dehydrogenase converts succinate to fumarate (Fig. 6). The fact that the *E. coli* CFT073 *sdhA* mutant generated about 2,000-fold fewer persister cells than *E. coli* CFT073 suggested the possibility that the ability to make fumarate from succinate was required for persister cell formation. To test that possibility, *E. coli* CFT073 *sdhA* cultures were grown overnight in liquid 0.4% glucose M9 minimal medium in the presence and absence of fumarate (200 µg/ml), and persister cell assays were performed in 0.2% glucose M9 minimal medium in the presence of fumarate for cultures grown with fumarate and in the absence of fumarate for cultures grown without fumarate. In the presence of fumarate and ampicillin, *E. coli* CFT073 *sdhA* cultures generated between 10^5^ and 10^6^ CFU/ml persister cells (Fig. 9), much like *E. coli* CFT073 (Fig. 4, 5, 7, and 8), whereas in the absence of fumarate and the presence of ampicillin, *E. coli* CFT073 *sdhA* cultures generated only 5 × 10^2^ CFU/ml persister cells (Fig. 9). Therefore, allowing completion of the TCA cycle by supplying *E. coli* CFT073 *sdhA* with fumarate rescued its ability to generate a high level of persister cells, suggesting that a complete oxidative TCA cycle is required for maximal persister cell generation.

**FIG 10:**
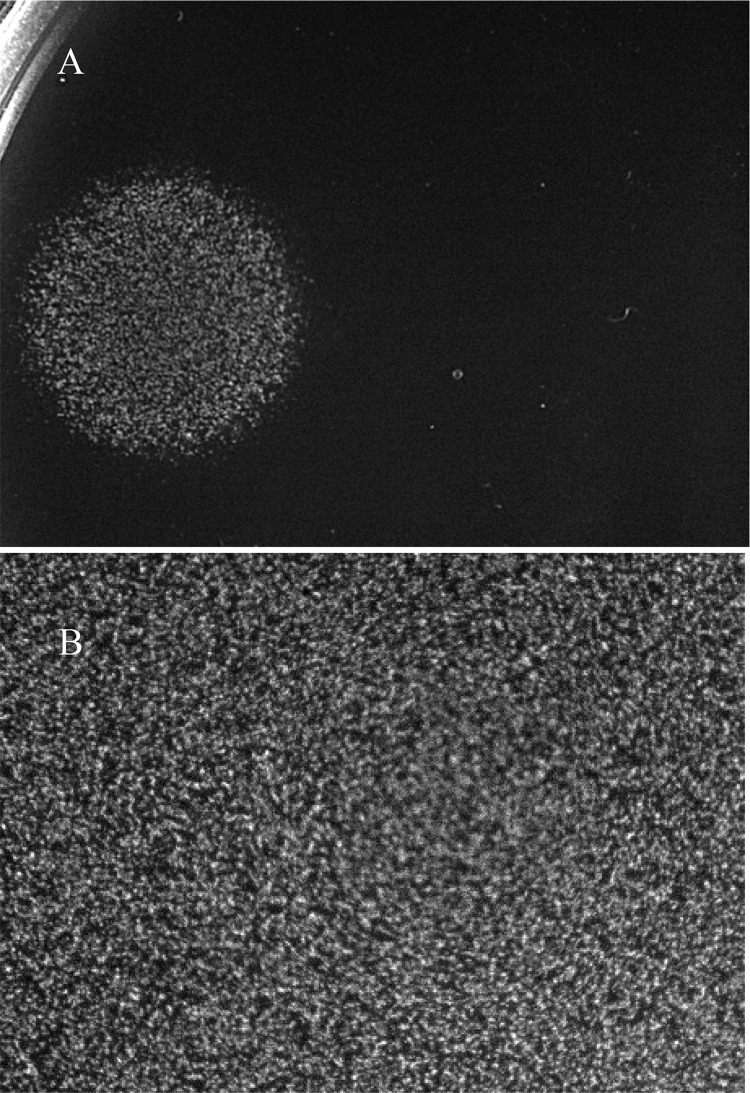
Rescue of *E. coli* CFT073 *sdhA* quiescence by fumarate. *E. coli* CFT073 *sdhA* was grown overnight in 0.4% glucose M9 minimal medium, and inocula of 10^5^ CFU were seeded on 0.2% glucose plates containing disodium fumarate (200 µg/ml) (A) or 0.2% glucose plates (B). One hour later, 5 µl of a mixture of 1.0 mM lysine, 1.0 mM methionine, and 1.0 mM tyrosine was spotted to each plate, and the plates were incubated at 37°C for 24 h.

Since persister cell generation required a complete TCA cycle, we wondered whether the same was true of quiescence. To that end, *E. coli* CFT073 *sdhA* was grown overnight in liquid 0.4% glucose M9 minimal medium in the presence and absence of fumarate (200 µg/ml). Glucose plates containing fumarate (200 µg/ml) were seeded with 10^5^ CFU of *E. coli* CFT073 *sdhA* grown in the presence of fumarate or 10^5^ CFU of *E. coli* CFT073 *sdhA* grown in the absence of fumarate and were incubated at 37°C for 24 h. In both cases, *E. coli* CFT073 *sdhA* failed to grow but responded to 5 µl of a 1.0 mM mixture of lysine, methionine, and tyrosine (Fig. 10A), suggesting not only that the presence of fumarate in the glucose plate rescued quiescence but that quiescence was generated on the glucose plate and not in the liquid culture. As a control, glucose plates without fumarate were seeded with 10^5^ CFU of *E. coli* CFT073 *sdhA* grown overnight in liquid glucose M9 minimal medium in the absence of fumarate and plates were incubated at 37°C for 24 h. As expected, a lawn of *E. coli* CFT073 *sdhA* growth was observed on the glucose plates under these conditions (Fig. 10B). Therefore, not only does fumarate rescue the ability of *E. coli* CFT073 *sdhA* to generate a high level of persisters in liquid glucose M9 minimal medium in the presence of ampicillin (Fig. 9), it rescues the ability of *E. coli* CFT073 *sdhA* to enter the quiescent state on glucose plates. As a side note, rescue by fumarate, obviating the need for succinate dehydrogenase, further implicates the mini-Tn*5* Km insertion and not a downstream polarity effect as the cause of nonquiescence and reduced persistence of the *sdhA* mutant.

### The *E. coli* CFT073 original clinical isolate is quiescent on glucose plates but generates very few persister cells in liquid glucose M9 minimal medium.

**FIG 11:**
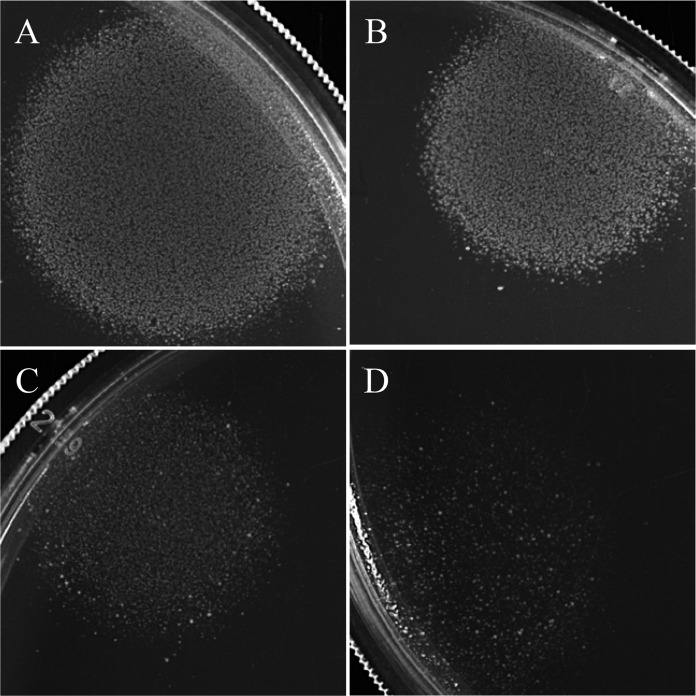
Quiescence of the *E. coli* CFT073 original clinical isolate on glucose plates. Glucose (0.2%) plates were seeded with 10^5^ CFU of the *E. coli* CFT073 original clinical isolate, and 5-µl amounts of the following mixtures (containing 1.0 mM of each amino acid) were spotted onto the plates: (A) lysine, methionine, and tyrosine; (B) lysine and methionine; (C) lysine and tyrosine; (D) methionine and tyrosine. The plates were incubated at 37°C for 24 h.

As we were completing this study, we became aware of a paper reporting that the sequenced *E. coli* CFT073 (22), which has long been considered to be the wild-type strain, has a 5-bp duplication in *rpoS* which results in a truncated, nonfunctional RpoS (34). RpoS, often referred to as the alternative sigma factor σ^S^, directs RNA polymerase to transcribe genes involved in the *E. coli* general stress response, e.g., acid resistance (35). The *E. coli* CFT073 strain used in this study does indeed have the 5-bp duplication in *rpoS* (M. P. Leatham-Jensen, unpublished results). We therefore obtained and tested the original clinical isolate of *E. coli* CFT073, which has a wild-type *rpoS* gene (34), for quiescence and persister cell formation. The *E. coli* CFT073 original clinical isolate is indeed quiescent on glucose plates and responds to lysine, methionine, and tyrosine like the *rpoS* mutant used here (Fig. 11), but it generates about 2,000-fold fewer persister cells in liquid glucose M9 minimal medium than the *rpoS* mutant used in the present study (Table 3). Therefore, it appears that the mutant *rpoS* gene is necessary for the high level of persistence observed but not for *E. coli* CFT073 quiescence on glucose plates.

## DISCUSSION

The data presented here show that the uropathogen *E. coli* CFT073 and the probiotic *E. coli* Nissle 1917, both ST73 strains belonging to phylogenetic group B2, are quiescent on glucose plates seeded with ≤10^6^ CFU, as are 35/45 (77.8%) additional ST73 strains tested. ST73 is a very common UPEC lineage (24, 25). In contrast, of 4 phylogenetic group A, 6 phylogenetic group B1, 6 phylogentic group D, 4 phylogenetic group ABD, and 4 phylogenetic group AxB1 strains, none (0/24) were quiescent. However, 9 of 40 randomly selected UPEC strains isolated from community-acquired UTIs in Denmark (22.5%) were quiescent on glucose plates (3/5 ST73 strains, 3/3 ST141 strains, 1/1 ST104 strain, 1/1 ST394 strain, and 1/1 ST998 strain). Thus, quiescence on glucose plates is common among UPEC isolates and is not restricted to one ST type.

The data presented here also show that the *E. coli* CFT073 original clinical isolate, like the *E. coli* CFT073 strain used here, which has a 5-bp duplication in *rpoS* that inactivates the gene (33), is quiescent on glucose plates (Fig. 11), but unlike the *E. coli* CFT073 strain used here, the *E. coli* CFT073 original clinical isolate generates a low level of persister cells in the presence of ampicillin (Table 3). Therefore, quiescence and persistence, while similar in some respects, i.e., both are prevented by amino acids and both require a complete oxidative TCA cycle, are not identical. In addition, quiescence and persistence differ in that of the 5 *E. coli* CFT073 mini-Tn*5* nonquiescent mutants isolated in the present study (*gdhA*, *gnd*, *pykF*, *sdhA*, and *zwf* mutants), only 2 (*gdhA* and *sdhA*) were deficient in persister cell formation (Table 3).

It has been reported previously, as reported here for *E. coli* CFT073, that deleting *rpoS* in *E. coli* K-12 also dramatically increases the formation of persister cells in the presence of ampicillin (36). Why then, does *E. coli* Nissle 1917, which has a wild-type *rpoS* gene (36; M. P. Leatham-Jensen, unpublished results), generate at least as high a level of persister cells as *E. coli* CFT073 (Table 3)? *E. coli* Nissle 1917 would be expected to be acid resistant, since a wild-type *rpoS* gene is required for acid resistance; however, it has been reported to be extremely acid sensitive (37). This suggests the possibility that the *E. coli* Nissle 1917 *rpoS* gene may be poorly expressed or that RpoS is rapidly degraded, which is consistent with its generating as high a level of persister cells as the *E. coli* CFT073 *rpoS* mutant used here. Many *E. coli* strains contain wild-type *rpoS* genes that are expressed poorly relative to the *rpoS* expression in other strains (38).

While it is unclear why *E. coli* CFT073 *gnd* and *zwf* mutants are nonquiescent on glucose plates, the reason why *E. coli* K-12 *gnd* and *zwf* mutants grow on glucose as the sole carbon source is known (29, 30). When *E. coli* K-12 is grown on glucose as the sole carbon source, glucose-6-phosphate dehydrogenase, encoded by *zwf*, and 6-phosphogluconate dehydrogenase, encoded by *gnd*, are used for the synthesis of ribulose-5-phosphate via the oxidative branch of the pentose phosphate pathway (Fig. 6) (28, 29). Ribulose-5-phosphate is an essential precursor in the synthesis of FAD, nucleotides, and LPS. Furthermore, both enzymes generate NADPH for biosynthesis. It might therefore seem surprising that mutations in *gnd* and *zwf* allow growth on glucose as the sole carbon source (Fig. 6). However, null mutations in *gnd* and *zwf* in *E. coli* K-12 do not significantly affect the growth of the mutants on glucose because the nonoxidative branch of the pentose phosphate pathway runs backwards in these mutants, generating ribulose-5-phosphate from fructose-6-phosphate and glyceraldehyde-3-phosphate (Fig. 6) (28, 29) and the necessary NADPH for biosynthesis by increased flux through the TCA cycle (28, 29). It is therefore possible that, for unknown mechanistic reasons, the oxidative branch of the pentose phosphate pathway operates minimally during quiescence for *E. coli* CFT073, generating little ribulose-5-phosphate, but that the *gnd* and *zwf* mutations cause the nonoxidative branch to run backwards and flux through the TCA cycle to increase, generating sufficient levels of ribulose-5-phosphate and NADPH to prevent quiescence. This scenario is consistent with the observation that ribose and xylose, both of which are metabolized in the nonoxidative branch of the pentose phosphate pathway, prevent quiescence when used as sole carbon sources (Fig. 2).

The *E. coli* CFT073 *pykF* mutant also grows on glucose as the sole carbon source. The *pykF* gene encodes pyruvate kinase, which converts phosphoenolpyruvate (PEP) to pyruvate (Fig. 6). There is a second pyruvate kinase, encoded by *pykA*, but during the growth of *E. coli* K-12 in glucose minimal medium, it contributes little to total pyruvate kinase activity (39). Moreover, in the complete absence of pyruvate kinase, pyruvate can still be generated in *E. coli* K-12 both through glucose transport via the phosphotransferase transport system (PTS) (40) and via the Entner-Doudoroff pathway (31, 38). Therefore, it is not surprising that the nonquiescent *E. coli* CFT073 *pykF* mutant can grow on glucose as sole carbon source, but why is it nonquiescent? It has been suggested that *pykF* mutants contain increased intracellular levels of PEP (30). If PEP, a common precursor in lysine, methionine, and tyrosine biosynthesis (Fig. 6), is increased in the *E. coli* CFT073 *pykF* mutant growing on glucose plates, sufficient intracellular levels of the 3 amino acids might be generated to stimulate growth and prevent quiescence.

The *gdhA* gene encodes glutamate dehydrogenase, which converts α-ketoglutarate to glutamate (Fig. 6). It is known that *E. coli* K-12 *gdhA* mutants grow on glucose as the sole carbon source in the absence of glutamate dehydrogenase because glutamate can also be synthesized via the sequential action of glutamine synthetase and glutamate synthase (31, 41). Therefore, it appears that the switch from producing glutamate via glutamate dehydrogenase to producing it via glutamine synthetase and glutamate synthase in *E. coli* CFT073 somehow prevents quiescence. It has been estimated that 88% of assimilated nitrogen in *E. coli*, including the nitrogen in amino acids, originates from glutamate, and the remaining 12% from glutamine (42). Perhaps glutamate and glutamine are in higher concentrations in *gdhA* mutants growing on glucose, which could contribute to higher intracellular levels of lysine, methionine, and tyrosine and, as a consequence, nonquiescence.

*E. coli* K-12 succinate dehydrogenase mutants grow on glucose as a sole carbon source because it is not necessary for the TCA cycle to function as a complete oxidative cycle to achieve growth (43). In fact, when growing on excess glucose, the *E. coli* K-12 TCA cycle operates in branched mode, i.e., an oxidative branch which runs from citrate to α-ketoglutarate and a reductive branch which runs backwards from oxaloacetate to succinyl-coenzyme A (CoA) (Fig. 6) (43). Succinate dehydrogenase is not necessary under these conditions, and both branches serve biosynthetic functions. As a result, neither branch is used for ATP generation (43). ATP is generated from glycolysis and via the phosphotransacetylase (*pta*)-acetate kinase (*ackA*) pathway, producing acetate in the process (Fig. 6) (43). Therefore, it appears likely that forcing the TCA cycle to operate in branched mode somehow prevents *E. coli* CFT073 quiescence. In branched mode, the TCA cycle is unable to regenerate oxaloacetate and therefore gets oxaloacetate from PEP via PEP carboxylase (Ppc) (Fig. 6). Perhaps under these conditions, sufficient oxaloacetate is generated via Ppc to increase the intracellular levels of lysine and methionine (Fig. 6) and, consequently, prevent quiescence. In this vein, the addition of fumarate to glucose plates results in the return of the *E. coli* CFT073 *sdhA* mutant to quiescence, presumably by rescuing the ability of the TCA cycle to operate as a complete oxidative cycle. It should also be noted that an *E. coli* CFT073 succinate dehydrogenase mutant (*sdhB*) has been shown to be severely attenuated in an ascending UTI mouse model (44), indicating a possible link between *in vitro* nonquiescence and reduced pathogenesis *in vivo*.

While it is clear why the *E. coli* CFT073 mini-Tn*5* Km nonquiescent mutants are capable of growth using glucose as the sole carbon source, it is not mechanistically clear why the mutations prevent quiescence on glucose plates. Possibly a gene encoding a regulator whose synthesis or activity is inhibited by various combinations of lysine, methionine, and tyrosine promotes quiescence. The regulator would presumably be expressed or active in the vast majority of *E. coli* CFT073 cells on glucose plates but in relatively few cells of the *E. coli* CFT073 mini-Tn*5* Km nonquiescent mutants due to the complex metabolic changes that occur in such mutants (28–30, 39, 45, 46). If the expression and activity of a specific *E. coli* CFT073 regulator is critical for the generation of quiescence, much as toxin/antitoxin systems appear to be critical in generating persister cells (11, 47), screening more mini-Tn*5* Km mutants may lead to its identification. Furthermore, it is unclear why the *E. coli* CFT073 *gdhA* and *sdhA* mutants generate far fewer persister cells than the *gnd*, *zwf*, and *pykF* mutants (Table 3). Metabolic flux analysis (28–30) and RNA-seq (48) may prove useful in this regard.

How might the findings reported here be relevant to recurrent UTI infections, and how might their relevance be tested? It is known that *E. coli* CFT073 utilizes amino acids and small peptides as carbon sources and a complete oxidative TCA cycle to infect the mouse urinary tract (44, 49), and it appears to import small peptides to grow in mouse urine *in vitro* (44). UPEC may also use peptides for growth *in vivo* in urine during human UTI (50). It is also known that *E. coli* CFT073 can infect mouse superficial facet cells and form intracellular biofilmlike communities (IBCs) (51) and, therefore, most likely can form quiescent intracellular reservoirs (QIRs) in underlying transitional cells. Furthermore, it seems reasonable that QIRs surviving antibiotic treatment serve as a reservoir for recurrent UTI infection after the withdrawal of antibiotics (8, 10), i.e., as transitional cells undergo apoptosis and released QIRs resume growth in urine, using peptides and amino acids in the process (44, 49, 50). However, it should be noted that the role of QIRs in recurrent UTI is still controversial. It has recently been shown that UPEC strains isolated from the feces and urine of female patients during recurrent episodes are identical, consistent with the possibility that recurrent UTI is caused by UPEC strains that colonize the intestine but periodically move to the urinary tract (52). Nevertheless, if the *in vitro* quiescence reported here mimics the QIR state and if QIRs are a major source of recurrent UTI, the *E. coli* CFT073 nonquiescent mutants we have isolated should be less able to establish QIRs in the mouse bladder and, therefore, be less able to cause recurrent UTI. If so, it is possible that drugs designed to inactivate the enzymes encoded by *gdhA*, *gnd*, *pykF*, *sdhA*, and *zwf* might be effective in limiting recurrent urinary tract infection. On the other hand, if the *in vitro* persistence state mimics the QIR state in mouse bladder cells, only the *E. coli* CFT073 *gdhA* and *sdhA* mutants should be less able to cause recurrent UTI, and if so, drugs designed to inactivate the enzymes encoded by *gdhA* and *sdhA* might be effective in limiting recurrent urinary tract infection A recurrent UTI mouse model is available for testing these hypotheses (8).

## MATERIALS AND METHODS

### Bacterial strains.

The *E. coli* CFT073, MG1655, and Nissle 1917 strains used in this study are listed in Table 1. The original *E. coli* K-12 strain was obtained from a stool sample from a convalescing diphtheria patient in Palo Alto, CA, in 1922 (12). The sequenced *E. coli* MG1655 strain (CGSC 7740) was derived from the original K-12 strain, having only been cured of the temperate bacteriophage lambda and the F plasmid by means of UV light and acridine orange treatment (12). *E. coli* Nissle 1917 was originally isolated during World War I from a soldier who escaped a severe outbreak of diarrhea (13). It has a beneficial effect on several types of intestinal disorders, is well tolerated by humans, and has been marketed as a probiotic remedy against intestinal disorders in several European countries since the 1920s (13). *E. coli* strains tested for quiescence as described in “Testing additional *E. coli* strains for quiescence on glucose plates” and “Testing additional ST73 strains for quiescence on glucose plates” in Results are from the E. V. Sokurenko collection, and the *E. coli* strains used as described in “Testing 40 UPEC strains isolated from community-acquired UTIs in Denmark for quiescence on glucose plates” in Results are from the N. Frimodt-Møller and K. L. Nielsen collection.

### Media.

LB broth (Lennox) (Difco Laboratories) and LB agar (Lennox) (Difco Laboratories) were used for routine cultivation. SOC medium was prepared as described by Datsenko and Wanner (14). Liquid M9 minimal medium (15) and M9 minimal medium agar plates were supplemented with reagent grade (Sigma-Aldrich, Inc.) l-arabinose (0.2%, wt/vol), *N*-acetyl-d-glucosamine (0.2%, wt/vol), l-fucose (0.2%, wt/vol), d-fructose (0.2%, wt/vol), d-glucose (0.2%, wt/vol), d-galactose (0.2%, wt/vol), d-gluconate (0.2%, wt/vol), glycerol (0.2%, vol/vol), d-mannose (0.2%, wt/vol), maltose (0.2%, wt/vol), d-ribose (0.2%, wt/vol), d-xylose (0.2%, wt/vol), potassium acetate (0.4%, wt/vol), sodium succinate (0.4%, wt/vol), and various amino acids, as indicated in Results. Since Difco Bacto agar contains impurities, M9 minimal medium agar plates were made with 1.6% Difco noble agar (Difco).

### Lawn assay for quiescence.

All *E. coli* strains were streaked from stored (−80°C) LB broth-grown cultures diluted 1:1 with 50% (vol/vol) glycerol to LB agar plates, which were incubated overnight at 37°C. A loopful of cells from each streak plate was then grown overnight in 10 ml of 0.4% glucose M9 minimal medium at 37°C with shaking in 125-ml tissue culture bottles. Routinely, 10^5^ CFU from an overnight culture was added to a tube containing 3 ml of liquid overlay medium (0.2% glucose M9 minimal medium, 0.7% Difco noble agar at 45°C). Each tube containing inoculated overlay medium was immediately poured onto a prewarmed (37°C) 0.2% glucose M9 minimal medium agar plate. Inoculated overlays were allowed to solidify (with lids slightly ajar) for 1 h at room temperature. In addition, as indicated, solidified inoculated overlays were stabbed with colonies of *E. coli* MG1655 grown on 0.4% glucose M9 minimal medium agar plates, using sterile toothpicks, or were spotted with 5 µl to 20 µl of filtered *E. coli* MG1655 culture supernatant, human urine, or defined amino acid solutions. Spots were allowed to dry prior to incubation of plates. Plates were incubated for 24 h or 48 h at 37°C, as indicated. Strains that grew as lawns on 0.2% glucose M9 minimal medium agar plates were considered to be nonquiescent, and strains that grew in liquid 0.2% glucose M9 minimal medium, failed to grow on 0.2% glucose M9 minimal medium agar plates, but were stimulated to grow around *E. coli* MG1655 stabs were considered to be quiescent (see Results).

### Insertional mutagenesis.

Mini-Tn*5* kanamycin (Km) mutants were constructed by insertional mutagenesis as described previously (16). Briefly, the donor strain *E. coli* ATM161 (17), carrying the suicide vector pUT, which contains the mini-Tn*5* Km transposon, was conjugated with the recipient strain *E. coli* CFT073 Str^r^ (Table 1) in the following manner. The donor and recipient strains were grown overnight, with shaking, in LB broth at 30°C. Aliquots of 100 µl of each culture were mixed together in 5 ml of 10 mM MgSO_4_ and filtered through a 0.45-μm-pore-size membrane filter (EMD Millipore). The filter was placed on the surface of an LB agar plate and incubated for 5 h at 37°C. Following incubation, the bacteria on the filter were suspended in 5 ml of 10 mM MgSO_4_, 100-µl aliquots of the suspension were plated on LB agar containing streptomycin sulfate (100 µg/ml) and kanamycin sulfate (80 µg/ml), and the plates were incubated for 18 h at 37°C. Individual mini-Tn*5* Km mutant colonies were transferred on toothpicks to 2 LB agar plates, one lacking and one containing ampicillin sodium salt (100 µg/ml; Sigma-Aldrich, Inc.). Colonies that were ampicillin sensitive, signifying loss of the pUT suicide plasmid, were transferred on toothpicks to sterile 16-mm-diameter culture tubes containing 250 µl of 0.2% glucose M9 minimal medium. The culture tubes were incubated overnight at 37°C with shaking, and then 5 ml of M9 minimal medium lacking a carbon source was added to each tube and 3 µl from each tube was spotted on a 0.2% glucose M9 minimal medium agar plate. The spots were allowed to dry (with lids slightly ajar), and the plates were incubated overnight at 37°C. Each spotted mini-Tn*5* Km mutant that grew was retested by seeding 10^5^ CFU of an overnight 10-ml 0.4% glucose M9 minimal medium liquid culture on a 0.2% glucose M9 minimal medium agar plate and incubating at 37°C for 24 h. The gene inactivated in each of the mini-Tn*5* Km mutants that grew as a lawn was determined by arbitrary PCR (18), as described below. In addition, to be sure that the mini-Tn*5* Km insertion was the cause of the ability of the individual mutants to grow on glucose plates, the insertion in each mutant was transferred into a fresh *E. coli* CFT073 Str^r^ background by the method of Wanner and Datsenko (14). Each mutant thus obtained was confirmed for the ability to grow as a lawn on glucose plates and for the position of the insertion within the *E. coli* CFT073 chromosome by both PCR and sequencing (Table 4 lists the primers used). Five confirmed mutants were isolated from approximately 2,000 mini-Tn*5* Km mutants tested.

**TABLE 4:** PCR primer sequences for amplifying mutant genes containing mini-Tn*5* Km insertions

Gene	Primer 1 (5′→3′)	Primer 2 (5′→3′)
*gdhA*	GATGGTCGAGTGGCAGATTAC	CAGAGGCTACTCAATGGCTTAC
*gnd*	GTTGGTTAAATCAGATTAATCCAGCC	CAACAGATCGGCGTAGTCG
*pykF*	CTGTAGCAATTGAGCGATGATG	ATCAGGGCGCTTCGATATAC
*sdhA*	CCGTTCCCATACCGTTTCTG	TTTCACCGGATCAACGTGAG
*zwf*	CCGGTAAAATAACCATAAAGGATAAGC	GAGAATGACATGGCGGTAAC

### Arbitrary PCR.

Arbitrary PCR was performed as described previously (18). Genomic DNA was isolated from *E. coli* CFT073 mini-Tn*5* Km mutants using the Wizard genomic DNA purification kit (Promega). The first round of PCR was performed in 25-µl reaction mixture volumes containing 1× standard *Taq* reaction buffer (New England Biolabs, Inc.), 2 mM deoxynucleoside triphosphates, 100 µM arbitrary primer 1 (5′ GGCCACGCGTCGACTAGTACNNNNNNNNNNNNGATAT 3′), 10 µM Tn*5*-specific primer (5′ TCTGGATTTCGATCACGGCACGT 3′), *Taq* DNA polymerase (2 units; New England Biolabs, Inc.), and DNA from 1.25 µl of an overnight LB broth culture. The first-round cycling conditions were as follows: (i) 4 min at 95°C, (ii) 6 cycles of 30 s at 95°C, 30 s at 30°C, and 1.5 min at 72°C 1.5, (iii) 30 cycles of 30 s at 95°C, 30 s at 45°C, and 2 min at 72°C, and (iv) 4 min at 72°C. The second round of PCR used standard conditions and cycling as follows: (i) 4 min at 95°C, (ii) 35 cycles of 30 s at 95°C, 30 s at 55°C, and 2 min at 72°C, and (iii) 4 min at 72°C, with 0.5 µl of the first-round PCR product as the template, 10 µM arbitrary primer 2 (5′ GGCCACGCGTCGACTAGTAC 3′), and 10 µM of a second Tn*5*-specific primer (5′ TTACCGAGAGCTTGGTACCCAGTC 3′). The second-round-PCR products were column purified with a QIAquick PCR purification kit (Qiagen) and sequenced using a third Tn*5*-specific primer (5′ GTACCCAGTCTGTGTGAGCAGG 3′). DNA sequencing was done at the URI Genomics and Sequencing Center, University of Rhode Island, Kingston, RI, using an Applied Biosystems 3130xl genetic analyzer (Applied Biosystems, Foster City, CA). A BigDye Terminator cycle sequencing kit (version 3.1; Applied Biosystems) was used for the sequencing reactions. The sequences were compared with the GenBank DNA sequence database using the BLASTX program.

### Identification and quantitation of amino acids in *E. coli* MG1655 and *E. coli* CFT073 50-fold-concentrated culture supernatants.

*E. coli* MG1655 was grown overnight in five 10-ml cultures in liquid 0.4% glucose M9 minimal medium with shaking at 37°C in 125-ml tissue culture bottles. The cultures were pooled, and cells were centrifuged for 10 min at 8,000 × *g* and resuspended in 1 ml of 0.2% glucose M9 minimal medium. The 50-fold-concentrated cultures were incubated standing overnight at 37°C in 1.5-ml centrifuge tubes (Celltreat Scientific Products). Cells were then centrifuged at 16,000 × *g* for 3 min, and the supernatant was removed and filtered free of bacteria using a 0.22-µm mixed cellulose ester (MCE) syringe filter (Fisherbrand). *E. coli* CFT073 concentrated culture supernatants were prepared identically.

The amino acids in 50-fold-concentrated culture supernatants were identified and quantified using a slightly modified version of a method described by Yuan et al. (19). The method uses the AccQ-Tag amino acid analysis method (Waters Corp., Milford, MA) with a pre-column derivatization kit (http://www.waters.com/webassets/cms/support/docs/wat0052881.pdf). Acid hydrolysis was not included in the derivatization step to ensure that only free amino acids were quantified. The method uses high-performance liquid chromatography (HPLC) to separate the derivatized amino acids and a fluorescence detector to identify and quantify them. The chromatographic method using AccQ tag eluent A (solvent A) and 60% acetonitrile in water (solvent B) was as follows: consecutive linear gradients of 0 to 2% B over 0.5 min; 2 to 7% B over 14.5 min; 7 to 10% B over 4 min; 10 to 20% B over 11 min; 20 to 36% B over 10 min; and 36 to 100% B over 5 min. The method totals 45 min of gradients and an additional 11-min washout phase. The flow rate was constant at 0.7 ml/min using a Waters AccQ Tag column (150 by 3.9 mm) held at a 40°C and injecting 5-µl aliquots. The fluorescence detector was set to an excitation wavelength of 250 nm and emission wavelength of 395 nm, according to the manufacturer’s instructions. Amino acids were identified by comparison to derivatized amino acid reference standards in the following order: tryptophan, 6-aminoquinoline (AMQ; a product of the derivatization process), aspartic acid, serine, glutamic acid, glycine, histidine, ammonium ion, arginine, threonine, alanine, proline, cysteine, tyrosine, valine, methionine, lysine, isoleucine, leucine, phenylalanine. A calibration curve was generated to quantify each amino acid present.

### Persister assay.

Overnight cultures in liquid 0.4% glucose M9 minimal medium were grown as described in “Lawn assay for quiescence” above. Cultures were then diluted 20-fold into 10 ml of fresh 0.2% glucose M9 minimal medium (*A*_600_ of 0.1, ~10^8^ CFU/ml) in both the presence and absence of ampicillin sodium salt (100 µg/ml; Sigma-Aldrich, Inc.). The cultures were incubated with shaking at 37°C, and viable counts on LB agar plates were determined at 2, 4, 6, and 24 h. To determine whether cells surviving at 24 h in the presence of ampicillin were persister cells or ampicillin-resistant mutants, 5-ml amounts were washed free of ampicillin by centrifuging at 16,000 × *g* for 3 min, washing cell pellets twice in 5 ml of fresh 0.2% glucose M9 minimal medium, and resuspending cells in 5 ml of LB broth. Each 5-ml culture was then grown for 2.25 h at 37°C with shaking in a 125-ml tissue culture bottle, at which time a sample was taken for viable count. Ampicillin sodium salt (100 µg/ml) was then added to each culture, and 4 h later, a sample was again taken for viable count assay on LB agar plates. Plates were incubated at 37°C for 18 h prior to counting.

### Photography.

Images of agar plates were made using a Bio-Rad Molecular imager Gel Doc XR+ system with Image Lab Software.

### Statistics.

The mean results and standard deviations of the data are presented in Table 4. The data in Fig. 4, 5, 7, 8, and 9 were compared by a two-tailed Student’s *t* test. *P* values of ≤0.05 were interpreted as indicating a significant difference.
